# How environmental cues shape human face value perceptions—an exploration based on grounded theory

**DOI:** 10.3389/fpsyg.2026.1712907

**Published:** 2026-08-06

**Authors:** Liming Chen, Changpeng Yang, Youyi Li, Yang Luo

**Affiliations:** 1 Al-Farabi International Business School, Al-Farabi Kazakh National University, Almaty, Kazakhstan; 2 Qian Dongnan Prefecture Traditional Chinese Medicine Hospital, Kaili, China; 3 Department of Education, General Graduate School, The Catholic University of Korea, Bucheon-si, Republic of Korea

**Keywords:** aesthetics, environmental cues, face value, grounded theory, multidimensional attractiveness, symbolic capital

## Abstract

A high level of physical attractiveness can confer significant economic resources and behavioural advantages in social development, leading to increasing attention on the ‘appearance economy’. While evaluations of attractiveness lack uniform standards, it is intriguing that the public exhibits a high degree of consensus regarding who qualifies as ‘highly attractive’. This phenomenon suggests an underlying, discernible logic within aesthetic judgements. Faced with the escalating prevalence of ‘appearance capitalism’, there is an academic imperative to provide a theoretical interpretation of ‘physical attractiveness’, particularly to clarify the underlying logic of its aesthetic judgments. To explore this logic, this paper employs a Grounded Theory methodology. Through in-depth interviews with 35 participants from diverse backgrounds and genders, and following a three-tiered analytical process of open, axial, and selective coding, the data were analysed and refined. Empirical analysis of the interview data reveals that the essence of ‘facial attractiveness’ lies in a symbolically represented system jointly constructed by multiple environmental factors. This system comprises three intertextual dimensions: (1) a *physiological environmental* representation system formed by biological genetic attributes; (2) a *material environmental* representation system embodied by economic capital; and (3) a *social environmental* representation system shaped by accumulated socio-cultural capital. Building upon these findings, this study constructs an environmental suggestion model within the aesthetic logic of ‘facial attractiveness’. An individual’s appeal extends beyond biological optimisation of facial features, necessitating the systematic integration of multidimensional environmental factors. This integration process, functioning as a mechanism for reproducing symbolic capital, requires the establishment of a comprehensive personal charm generation system. This system operates through the three intertextual dimensions identified in this research. This theoretical framework offers a novel analytical perspective for understanding aesthetic capitalisation in contemporary society. It further establishes a conceptual model for formulating individual attractiveness management strategies.

## 1 Introduction

In recent years, an appearance-centred consumer culture has become deeply intertwined with consumerism, gradually evolving into a social trend sweeping across numerous countries worldwide. An attractive face often facilitates greater employment opportunities (Duan, 2014), earns others’ trust (Qi et al., 2018), and even enhances entrepreneurial success (Jiuli and Yuan, 2018). Appearance has become an implicit personal asset. As revealed by Hamermesh (2011) in his work *Beauty Pays*, which introduced the concept of ‘beauty economics’, the face has evolved into a form of capital that can be invested, utilised, monetised, and accumulated. Building upon this, Sarpila et al. (2020) further conceptualised this “facial capital” as ‘aesthetic capital’.

So, does a beautiful face truly hold value? Around 2015, the term ‘face value’ emerged and rapidly spread within China’s idol fandom circles, used to praise celebrities’ appearances. The American writer Whitefield-Madrano (2016), in her work *Face Value: The Hidden Ways Beauty Shapes Women’s Lives*, similarly employs ‘face value’ to explore how beauty moulds women’s lives. The popularity of ‘face value’ confirms a central proposition: an attractive face does indeed possess social value.

With the rapid development of the internet, traffic-driven economic models reliant on monetizing appearance have proliferated, driving the global expansion of ‘capitalisation of looks’. Empirical research confirms that social media use significantly increases adolescents’ and young adults’ willingness to invest in appearance while heightening their inclination toward facial cosmetic procedures (de Vries et al., 2014; Walker et al., 2021). Driven by both consumerism and visual culture, facial aesthetic features have been transformed into quantifiable symbolic capital, forming a unique ‘appearance-capital’ exchange mechanism. This mechanism not only reconfigures the logic of social resource allocation but also fosters a new economic paradigm centred on the medical aesthetics industry. This developmental trajectory reflects the aesthetic alienation prevalent in contemporary society (Chen, 2020).

The phenomenon of social capital premium derived from the symbolic power based on facial aesthetics is systematically giving rise to the ‘capitalisation of looks’, a typical symptom of postmodern societies—high-value individuals construct significant competitive advantages in the labour market, the competitive field of marriage, and the digital platform economy through the symbolic performance of their body habitus (Wei and Sun, 2019; Liu et al., 2016). The logic of its operation presents three significant features: firstly, there is a significant positive correlation between appearance advantage and income growth (Harper, 2000), which has been repeatedly verified in cross-country comparative studies (Hamermesh and Biddle, 1994), and ‘good image temperament’ as an implicit competence indicator forms a conjugate screening system with academic capital (Rhode, 2014); secondly, in the marriage market, facial features become important signals of choosing a spouse, which generates the effect of ‘face value’; finally, in the digital economy, looks are alienated into ‘traffic currency’, and capital appreciation is realised through new types of business, such as the live broadcasting economy (Wang, 2016).

It is worth noting that the development of this kind of ‘appearance capitalism’ presents the typical characteristics of ‘McDonald’s’: the aesthetic standard is becoming increasingly homogeneous, the evaluation system is constantly quantified, and the reproduction process is highly industrialised. Social media platforms reinforce the cognitive paradigm of ‘face value is justice’ through algorithmic recommendation, while medical aesthetic institutions transform this paradigm into a replicable business model through standardised services (Chen, 2016). 2016’s widely circulated online discourse of “relying on one’s face to eat” is not only a kind of playful expression, but also profoundly reveals the cognitive tendency of contemporary society to regard appearance and human capital as equal, which signals the formation of a new type of social stratification—the formation of a new social stratification based on appearance and human capital. In the face of this intensifying social phenomenon, it is imperative for academics to theorise and explain the concept of ‘face value’, especially to clarify the internal logic of its aesthetic judgment. This study aims to systematically analyse the influencing factors of ‘face value’ evaluation and reveal the cognitive mechanism behind the public aesthetic consensus, so as to provide a new theoretical perspective for understanding the phenomenon of face capitalisation in contemporary society.

## 2 Literature review

### 2.1 Conceptual genealogy and semantic evolution of ‘face value’

As an emerging sociological term, the conceptual connotation of ‘face value’ has undergone a complex semantic transmutation process. From the etymological point of view, the character ‘Face (颜)’ inherits the classical connotation of appearance in Chinese, while ‘Value (值)’ introduces the quantitative thinking in the context of modernity, and the combination of the two forms the unique cognitive paradigm of ‘the numerical value of appearance’. The Dictionary of Modern Chinese (7th edition, 2017) defines it as “the numerical value for evaluating a person’s appearance”, an official definition that reveals the cognitive shift in contemporary society toward the inclusion of physical features in the quantitative assessment system. In the academic discourse system, the concept of ‘face value’ presents a multidimensional theoretical tension. Under the theoretical framework of body aesthetics, Wang (2017) explicates it as a comparative measurement-based assessment system of looks.

It is noteworthy that the term ‘facial value’ gained popularity around 2015, undergoing a marked semantic broadening in the process: from initially denoting an assessment metric specifically for facial features, it gradually expanded to encompass a comprehensive evaluative dimension covering the appearance of objects, environmental characteristics, and even abstract attributes (Zhang, 2021; Hu, 2019). For instance, a mobile phone or computer with a higher value can be sold at a higher price, and “food with a high ‘face value’ can bring people a pleasant mood and appetite (Pfeiffer et al., 2021), whereas consuming visually unappealing food may heighten the risk of food poisoning” (Castagna et al., 2021). This semantic expansion reflects the deep cultural logic of ‘visual centrism’ in contemporary society, i.e., all existences can be included in the cognitive paradigm of aesthetic quantitative assessment.

### 2.2 Logic of judgement and extension of scope of ‘face value’

At the beginning, ‘face value’ was simply a description of people’s external appearance, generally used only for those with good looks. In the sentence “Beginning with ‘face value’, trapped in talent, loyal to the character,” ‘face value’ specifically refers to people’s faces (Yu et al., 2019). From the empirical research of social psychology, the level of ‘face value’ is significantly positively correlated with the degree of emotional arousal of the observer, and individuals with high ‘face value’ can not only induce stronger visual attention, but also produce pleasurable emotional experiences through the mechanism of aesthetic empathy, which then triggers convergent behavioural intention. This psychological effect essentially stems from the core concept of ‘attractiveness’, which is a comprehensive psychological trait of the target object that stimulates a positive emotional response from the observer and facilitates the willingness to interact with the observer. Based on this, academics have constructed a three-dimensional ‘face value’ evaluation system: (1) *facial attractiveness*, which refers to the aesthetic pleasure and social proximity effects of facial morphological features through visual perception, and is essentially a symbolic presentation of facial aesthetic features (Tatarunaite et al., 2004); (2) *physical attractiveness*, which extends the scope of evaluation to include biomorphological indicators such as body size index and expression of secondary sex characteristics, reflecting the adaptive assessment mechanism under the perspective of evolutionary psychology (Sugiyama, 2015); and (3) *overall appearance attractiveness*, which serves as an integrative dimension of the first two and initially constitutes the overall image of people’s ‘face value’. In terms of research methodology, a large number of researchers have used interviewer ratings of static pictures of people to differentiate between high and low personal ‘face value’ (Hies and Lewis, 2022; Kenrick and Gutierres, 1980). The aesthetic logic of human ‘face value’ originates from the stereotype of ‘good’ (Downs and Harrison, 1985).

Since the emergence of anthropology of the human body, interdisciplinary perspectives have been used to explore the biological determinants of facial attractiveness. Through computer-assisted measurements and biometric analyses, researchers related to medical aesthetics have established a statistically significant system of facial aesthetic parameters (Bashour, 2006). Beyond facial features, bodily characteristics also play a pivotal role in assessments of attractiveness. Singh (1993) research highlighted the significant aesthetic importance of the waist-to-hip ratio (WHR), explicitly proposing that female bodies with a WHR of 0.7 are perceived as most attractive. Building upon this foundation, Naor-Ziv et al. (2025) further enriched this perspective by discovering that the most appealing female body characteristics combine a low WHR with a high buttock-to-hip ratio (BHR). Hübner and Ufken (2024) contends that, compared to the singular WHR metric, the body’s overall curvature (i.e., BWR) more effectively predicts female physical attractiveness, aligning with Naor-Ziv et al.’s perspective. Consequently, some individuals opt for ‘Ant Waist Surgery’ to achieve their ideal WHR, involving the removal of the eleventh and twelfth ribs to sculpt the desired waistline (Chiu et al., 2023). Brierley et al. (2016) noted that Body Mass Index (BMI) and its implied muscularity significantly influence human attractiveness, a view supported by Han et al. (2023). The latter further discovered that men’s preferred BMI characteristics may signal ‘youthfulness’. Interestingly, research indicates that facial attractiveness is also influenced by genetics, such as the positive correlation between Major Histocompatibility Complex (MHC) diversity and male facial attractiveness (Hakkarainen et al., 2021). Clinical practices within the aesthetic medicine field suggest that the biological dimensions of facial attractiveness primarily encompass elements of: symmetry (Penton-Voak et al., 2001), sexual dimorphism (Little et al., 2011; Jones and Jaeger, 2019), neoteny, and other evolutionary psychological indicators (Fink and Penton-Voak, 2002). This bioaesthetic paradigm has fostered global aesthetic convergence, with notable variations observed only within certain subcultural groups such as ‘punk’ or ‘gothic’ (Kuipers, 2015).

In addition to biological traits, some non-biological traits have also gradually become factors influencing the ‘face value’ of human beings; Blount-Nuss et al. (2006) found that men in military uniforms were more attractive than men in civilian clothes. Meanwhile, certain sportswear brands have begun to place excessive emphasis on women’s gendered characteristics (Brice et al., 2023) rather than functionality. For instance, numerous prominent athletic apparel labels increasingly favour highlighting specific female body parts in their product promotions. Such marketing strategies readily contribute to the objectification of women (Hollett et al., 2022). Research indicates that clothing colour also influences women’s body perception, with black and red yielding higher physical attractiveness ratings while simultaneously creating a slimmer appearance (Sidhu et al., 2021). It is not surprising that adornment has been used to enhance personal attractiveness; people wear beautiful adornments, which enhance their attractiveness (Bloch and Richins, 1993). Clothing, hairstyles, make-up, facial hair, and accessories have been used as a non-verbal communication tool to convey certain messages about the wearer (Damhorst, 1990), and jewellery shops spend tens of thousands of dollars a year on promotions to convince consumers that adornments will give them a prestigious appearance and enhance a sense of well-being (Quarantelli, 1967; Bloch and Richins, 1992). Such adornments are gradually transformed into social status or aesthetic capital in changing socio-cultural contexts, and the aesthetic logic of ‘face value’ is gradually extended to the socio-cultural or material level. As a result, some sociologists and the media have argued that ‘aesthetic capital’—derived from the social status conferred by physical attractiveness—is a form of symbolic capital (Anderson et al., 2010).

Past research has shown that physically attractive and unattractive people may internalise different social images or interpersonal styles, and that social appraisal, social communication, internalised personality, and social behaviours influence a person’s attractiveness (Adams, 1977). It can be seen that ‘face value’ has been given new interpretations in the process of application and development, with the meaning of ‘value’ gradually weakening and the meaning of ‘face’ becoming more prominent. The evaluation of ‘face value’ focuses not only on individuals’ appearance but also extends to their deeper spiritual qualities. For example, the description of “the beauty of a beautiful man is in his bones” reflects a person’s ‘spiritual face value’, which is a deeper level of ‘face value’ (Chen, 1999). This hierarchical evaluation structure essentially maps the deep cognitive mechanism of human beings that couples sensory experience with value judgment.

Research on ‘face value’ has primarily centred on evolutionary psychology (Singh, 1993; Penton-Voak et al., 2001; Tatarunaite et al., 2004) and medical bioaesthetics (Bashour, 2006; Chiu et al., 2023; Han et al., 2023; Hakkarainen et al., 2021), predominantly employing experimental methods or biometric analysis (Bashour, 2006). These studies have examined body curves (Hübner and Ufken, 2024), BMI indices (Brierley et al., 2016; Han et al., 2023), facial symmetry (Penton-Voak et al., 2001), sexual dimorphism (Little et al., 2011; Jones and Jaeger, 2019), neoteny (Fink and Penton-Voak, 2002), and WHR (Naor-Ziv et al., 2025).

Some scholars have examined how clothing (Blount-Nuss et al., 2006; Brice et al., 2023), accessories (Damhorst, 1990; Quarantelli, 1967; Bloch and Richins, 1992), and facial structure (Bashour, 2006) significantly influence ‘face value’ assessments, yet the underlying psychological logic and specific mechanisms through which these factors affect attractiveness judgments remain unsystematically elucidated. Consequently, this study employs a Grounded Theory methodology. Through in-depth interviews and open coding, it systematically extracts the psychological processes by which diverse environmental factors influence ‘face value’ evaluations. It delves into the formation mechanisms of ‘face value’ attractiveness, constructing an environmental implication model in the logic of ‘face value’ aesthetics to reveal its underlying influence mechanisms. This provides a novel theoretical perspective for research on ‘face value’ attractiveness.

## 3 Data sources and research methodology

### 3.1 Research methods

In this study, an exploratory research technique, Grounded Theory (Charmaz, 2014; Glaser and Strauss, 1967), was employed to construct factors affecting personal attractiveness through three standard steps of open coding, spindle coding, and selective coding of textual data semantics. The strategy of continuous comparison is used in the process of data refinement to continuously refine the theory until the newly acquired data coding no longer contributes to the construction of the theory, i.e., the theory is saturated. Therefore, this study adopts the research method of Grounded Theory in-depth interviews and applies Nvivo12 software to process the raw data.

### 3.2 Data sources

The aesthetic judgment of ‘face value’ constitutes a socio-psychological phenomenon characterised by relative subjectivity and the absence of uniform measurement standards. Given that traditional quantitative scoring mechanisms struggle to reveal the intricate psychological processes and latent cognitive biases underlying individual aesthetic behaviour, qualitative research methodologies demonstrate superior suitability for addressing such issues. Among these, Grounded Theory—as a systematic theoretical construction method—is particularly well-suited to distilling the intrinsic logic and interpretative frameworks governing ‘face value’ assessments from empirical data. Consequently, this study employs a Grounded Theory as its primary methodology, utilising in-depth interviews with participants to uncover genuine thoughts and cognitive processes during ‘face value’ evaluations.

Regarding sample selection, the research primarily utilised random sampling supplemented by purposive sampling, aiming to encompass diverse occupational groups and gender categories to ensure representativeness and heterogeneity. Furthermore, considering the maturity of aesthetic cognition and linguistic independence, all participants were adults aged 18 years or older. Random sampling facilitates the accurate representation of aesthetic perceptions and attitudes within the general populace during daily life. To further obtain professional perspectives on ‘face value’ aesthetics from beauty industry practitioners, the research team randomly selected three medical aesthetics professionals at their workplace locations for interviews, based on interview convenience. The sample size was determined by the principle of theoretical saturation. After interviews with 31 participants, data coding yielded no additional core concepts or categories. To confirm theoretical saturation, the research team conducted supplementary interviews with four randomly selected participants. This confirmed sufficient theoretical saturation, rendering further sample expansion unnecessary (participant demographics are presented in Table 1 below).

**Table 1:** Basic information of respondents.

Respondent code	Age	Occupation	Interview format	Respondent code	Age	Occupation	Interview format
M-01	32	Self-employed	Individual Interview	M-19	63	Retired	Focus Group
F-02	28	Office Worker	Individual Interview	F-20	34	Kindergarten Teacher	Focus Group
M-03	45	Taxi Driver	Individual Interview	M-21	44	Property Manager	Focus Group
F-04	51	Retired	Focus Group	F-22	31	University Teacher	Focus Group
M-05	24	University Student	Focus Group	M-23	48	Automobile mechanic	Individual Interview
F-06	22	University Student	Focus Group	M-24	57	Farmer	Individual Interview
M-07	38	University Teacher	Focus Group	F-25	23	Office Worker	Individual Interview
F-08	19	University Student	Individual Interview	M-26	39	Medical Aesthetics Director	Individual Interview
M-09	29	Delivery Person	Individual Interview	F-27	41	Restaurant Owner	Focus Group
F-10	33	Bank Clerk	Individual Interview	M-28	27	Master’s Student	Focus Group
M-11	47	Government Employee	Focus Group	F-29	35	Medical Aesthetics Nurse	Focus Group
F-12	42	Hospital Doctor	Focus Group	M-30	29	Fitness Trainer	Focus Group
M-13	55	Security Guard	Focus Group	F-31	24	University Student	Individual Interview
F-14	26	Office Worker	Focus Group	M-32	46	Civil Servant	Individual Interview
M-15	41	Medical Aesthetics Employee	Individual Interview	F-33	38	Nurse	Individual Interview
F-16	37	Accountant	Individual Interview	M-34	32	Driver	Individual Interview
M-17	52	Factory Worker	Individual Interview	F-35	27	Administrative Assistant	Individual Interview
F-18	29	Salesperson	Individual Interview				

**Figure 1:**
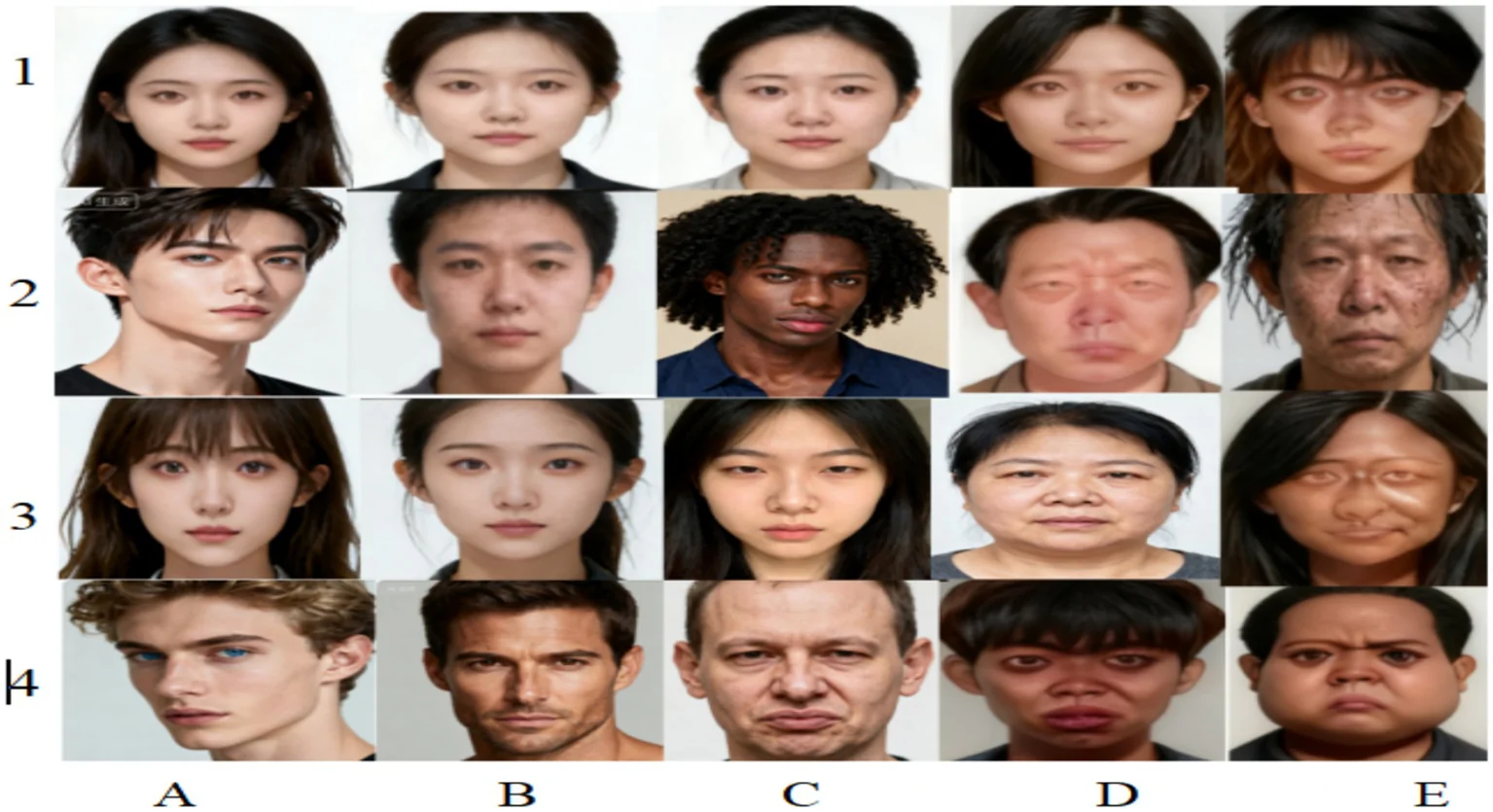
Personal face interview gallery. Figure contains images of the author(s) only.

**Figure 2:**
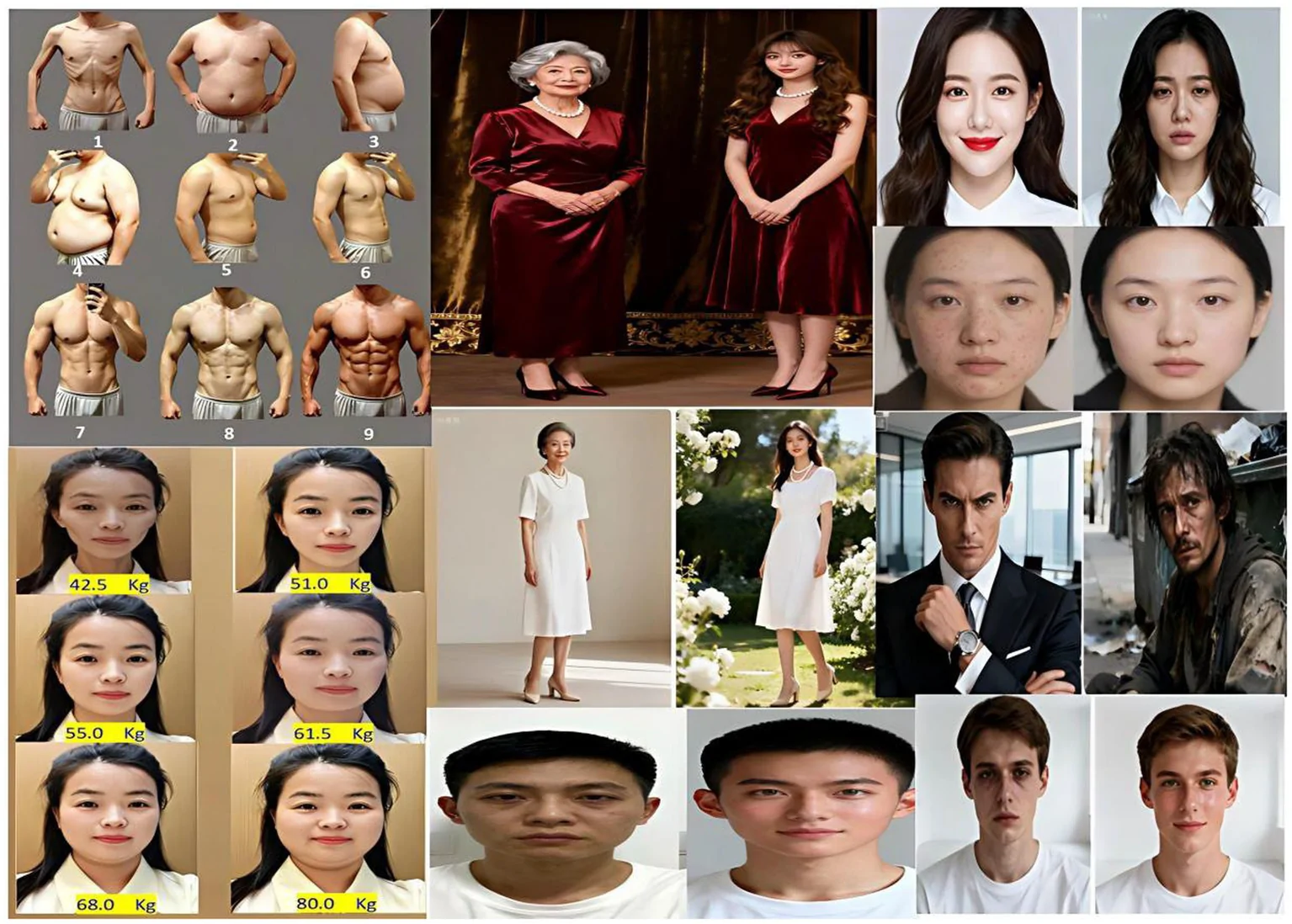
Comprehensive personal appearance interview gallery. Figure contains images of the author(s) only.

This study employed semi-structured in-depth interviews to ensure comprehensive exploration of the research themes. The interviews comprised two principal components:

Firstly, respondents were presented with 10 images of human facial features as visual stimuli (see Figures 1, 2). All facial profiles were randomly generated by artificial intelligence, aiming to showcase diversity in facial characteristics and body types without prescribing any specific ‘attractiveness’ criteria. Figure 1 focused on facial feature preferences, requiring participants to select faces they found attractive from grids A1 to E4 and articulate their reasoning. Figure 2 comprised nine photographs examining multi-dimensional aesthetic preferences, including body shape, perceived mental state, and age perception (e.g., preferences for youthful versus mature appearances with identical clothing conditions). To ensure the validity of aesthetic judgements, Figure 2 employed an intra-ethnic comparison logic. Comparisons within each dimension were confined to the same ethnic group to avoid potential ‘in-group preference’ biases arising from cross-ethnic comparisons.

Subsequently, the interview transitioned to an open-ended dialogue phase, delving into how participants form judgments about others’ appearances in daily life. The discussion centred on whether factors beyond physical features influence perceptions of attractiveness. Interview topics encompassed preferences for facial traits, perceptions of physical health, clothing styles, symbols of wealth, social status, personal talents, and character reputation, systematically exploring the multifaceted influences shaping ‘face value’ aesthetics.

The interview process employed both one-on-one in-depth interviews and group brainstorming sessions. Nineteen individuals participated in the one-on-one interviews, while four groups underwent the brainstorming format. Each group comprised one facilitator and four participants, with individual sessions lasting 2–3 h. The rationale for combining one-on-one in-depth interviews with group brainstorming sessions lies in the distinct advantages of each approach: one-on-one interviews enable participants to express their views freely, without concern for others’ opinions, allowing them to articulate their genuine thoughts without reservation. The group brainstorming approach, meanwhile, facilitated discussion and mutual inspiration among participants, encouraging respondents to articulate their genuine thoughts. This approach provided deeper insight into the logic people employ when judging ‘face value appeal’.

### 3.3 Ensuring rigor and trustworthiness of the study

To ensure the rigour and credibility of this Grounded Theory study, the entire research process strictly adhered to established protocols for qualitative research (Charmaz, 2014; Glaser and Strauss, 1967). First, theoretical saturation was adopted as the core criterion for determining the termination of data collection (Glaser and Strauss, 1967; Hennink et al., 2020). Data collection and open coding were conducted iteratively and concurrently—after each interview, the transcribed text was verified by the interviewee and immediately coded, and the constant comparative method was applied to compare new data with existing concepts and categories. Following 31 initial interviews, no new core concepts, categories or theoretical dimensions emerged from the coding process; after an additional four supplementary interviews, no new themes arose, indicating that full theoretical saturation had been achieved and that the data were sufficient to represent the phenomenon under investigation (Hennink et al., 2020). After all interviews were completed and theoretical saturation was confirmed, the researchers comprehensively and systematically organised, cross-checked, and verified all interview data, ultimately generating a complete interview record of over 100,000 words. This not only ensured the integrity and standardisation of the data but also laid a solid foundation for subsequent coding and analysis.

Second, triangulation was employed to enhance the validity of the study and reduce systematic bias (Denzin, 2009; Flick, 2018), which specifically included data triangulation (the cross-validation of data from different interviewees and contextual sources) and investigator triangulation (independent coding and cross-verification of results by multiple researchers to improve the accuracy and consistency of coding outcomes). Throughout the entire coding process, the researchers continuously compiled analytic memos to systematically document the emergence of concepts, the relational connections between categories, theoretical evolution, and coding reflections, thus forming a complete and verifiable audit trail to enhance the transparency and traceability of the analytical process. The constant comparison of raw data with emerging categories ensured that all research interpretations were grounded in empirical evidence rather than in subjective preconceptions, further enhancing the credibility and consistency of the analytical findings.

### 3.4 Data encoding

#### 3.4.1 Primary coding

Open Coding—at this stage we imported the raw interview data into NVivo 12 as the foundation for analysis. Employing a sentence-by-sentence approach, we systematically organised the textual content to extract meaningful units pertinent to the research theme. Operationally, we first focused on labelling keywords and key sentences that were closely related to the theme. To minimise potential subjective bias during coding, we endeavoured to derive labels directly from interviewees’ original statements, ensuring codes remained faithful to the original context. Building upon this, we further compared and synthesised the attributes and dimensions inherent in the primary labels, categorising and integrating concepts with similar meanings to ultimately form more generalisable categories. For instance, the raw data in Table 1—“B2’s skin condition is significantly better, smooth and fine-textured, appearing very healthy”—was labelled as “Good skin indicates good health,” with the corresponding sub-category being “Youthful Health Phenomenon.” This process achieved conceptualisation and categorisation of the initial material, laying the analytical foundation for subsequent theoretical construction (as shown in Table 2).

**Table 2:** Open codes.

Raw data and labelling	Generalization (subcategories)
*B2’s skin condition is significantly better, smooth and fine-textured, appearing very healthy (good skin indicates good health)*. That kind of *healthy complexion* gives a refreshing and clean feeling. Fine-textured, healthy skin makes a person look very pleasant and comfortable to be around.” (People with good skin have a healthy appeal.)	Young Health Phenomenon
“I appreciate *healthy, energetic body types* more, such as well-proportioned or athletic builds. This usually indicates that the person has *good lifestyle habits (having a healthy lifestyle)*, likely exercises regularly, possesses *better physical stamina*, and can withstand high-intensity work. Being excessively thin or obese may raise concerns about their *health status*.” (A healthy body and good living habits can make a good impression on others).	
“People with *fair, clean skin* appear very tidy, suggesting their *home hygiene and material conditions* are probably good. People with *acne* might carry parasites, bacteria, or viruses.”(*Health risk concerns)*	
“Individuals with *severe acne, skin ulcers, or numerous scars* make me feel apprehensive (Unhealthy traits make people afraid.). Since I am uncertain about the cause of their skin condition, I worry about *potential contagion*, which makes me reluctant to approach them.” (Avoiding contact with individuals posing a risk of infectious disease transmission)	Pathological risk aversion
“People with *facial disfigurements* evoke fear in me, primarily due to concerns about *being harmed or disfigured myself*. (Disfigurement evokes associations with unsafe environments). While I do not discriminate against them, I often perceive their *living environments as relatively unsafe*. Specifically, individuals with numerous *burn, knife, or gunshot wounds* tend to give me the impression of being prone to physical altercations.” (Violence conjures associations of unsafe environments)	Pathological risk aversion
“In a university setting, where frequent interaction with students and colleagues is necessary, individuals who are *healthy, young, and energetic* naturally foster a greater sense of *rapport and approachability.”* (Healthy people have a sense of closeness).	Pathological risk aversion
“People with *facial or other abnormal features* make me feel uncomfortable (psychological discomfort arising from deformity). I cannot explain why, but I *subconsciously sense fear* in my heart. “(An instinctive fear of deformity)	Pathological risk aversion
“I like people with *well-defined and exquisite facial features*. Perhaps they have a *stronger genetic expression ability*, and people with exquisite and beautiful facial features always look very healthy (Genetic advantage is more appealing). A beautiful face can also bring me a *pleasant feeling*, so I have a desire to get close to them.” (Health brings aesthetic pleasure)	Gene expression advantage
“I think features such as *big eyes, high nose bridge, and double eyelids* are more attractive because they make a person look more refined. However, I also think these features might be related to health. For example, *big eyes usually indicate good eye health*, and *a high nose bridge might be related to a good respiratory system.”* (Refined facial features may be linked to health or genetics)	Gene expression advantage
“I prefer *tall people*. Tall girls tend to have an *elegant appearance (tall people are attractive)*, and tall boys tend to be more *generous and give a sense of security*.” (Being tall gives a sense of security)	Gene expression advantage
“If there are women in *sexy clothes* on the street, I cannot help but look at them for a while longer. Their *body shapes and appearances* are all very attractive. Seeing beautiful women always gives me a comfortable feeling. “(Seductive attire evokes visual pleasure)	Symbolic dressing
“People who wear *expensive brand jewelry, watches, and clothing* seem to give off a sense of being wealthy. This indirectly shows that they have *a lot of social resources and material wealth*. They might have more ability to obtain resources than others, but this does not completely mean they possess good personal qualities.” (Material wealth influences personal attractiveness)	Symbolic dressing
“Yes, seeing a male in *military uniform* makes me feel particularly safe. The military uniform can particularly highlight their body shapes. They stand with a *straight posture (a good figure is very attractive)*. They exude an air of *justice and bravery*, and are very disciplined, which makes them very likable.”(The potential for inherent justice holds an appeal)	Symbolic dressing
“*Rich people* give off a different impression. Perhaps the ability to accumulate wealth gives people a *sense of security*, and it also indicates that they have *better access to social resources* and are more appreciated.” (Wealth and personal capability provide psychological security)	Resource intergenerational transmission
“When I see someone driving a *luxury car*, wearing a *name-brand watch or precious jewelry*, I think this person is quite capable. But this does not change my opinion of them. I value a person’s inner qualities and character more.” (Personal qualities are more appealing)	Resource intergenerational transmission
“I prefer those who *become rich through their own efforts* (Those possessing the quality of diligence are more appealing). Although the *second-generation rich* have good conditions, those who strive on their own have a greater sense of achievement. I think such people are more charming because they obtain their wealth through their own hard work.” (Those possessing the quality of diligence are more appealing)	Resource intergenerational transmission
“*Status* does indeed give a certain aura. If someone knows that he is a *doctor or a professor (Social* Status and the Cult of Competence), it will change people’s perception of him. For example, *Mr. Yuan Longping. He* is the most handsome scientist in my heart. “(Social Status and the Cult of Competence)	Social class and evaluation indicators
“If one wants to become a certain kind of person, one should be with people of that kind. Spending more time with people from *higher social classes* is likely to help change oneself (I hope to associate with outstanding individuals to transform myself). Spending too much time with lazy and bad people can also easily influence one. Some people, even if they have good looks, may still be rejected. For instance, certain celebrities who are in *prison for crimes* are also *despised by others.”* (Diminished moral character reduces goodwill)	Social class and evaluation indicators
“A person with *high social status* is truly very charming (Social Status and the Cult of Competence). This kind of “attractiveness” has nothing to do with good looks. Because humans are naturally drawn to *strong individuals (a tendency to idolise those who are strong)*, especially those who possess abilities that we do not have and can promote social progress. *Scientists* can explore the mysteries of the universe and develop life-saving drugs; *outstanding leaders* can influence the destinies of thousands of people. This immense creativity and influence will bring an extreme psychological impact, making us feel *admiration and even shock*. They will make us have the *desire to follow them.”(a tendency to idolise those who are strong)*	Social class and evaluation indicators
“Actually, when I first saw *pianist Lang Lang*, I thought he looked a bit oily and untidy.” But when I saw him playing “The Bell” on the piano bench, I felt his composure while *enjoying the performance*, which made me think *he was extremely cool.”* (a tendency to idolise highly skilled professionals)	Demonstration of capability signals
“My colleague Liu from the company taught me the techniques for *repairing cars*. His *skills are excellent,* and everyone respects him.” (a tendency to idolise highly skilled professionals)	Demonstration of capability signals
“I have a friend who is a *doctor*. He is extremely busy with his work, but every time I see him, I can feel his *professionalism and seriousness*. Such people look very pleasing to the eye.” (Responsible and capable individuals are attractive)	Demonstration of capability signals
“I have a senior who has *excellent grades, can play the piano, and won the first prize in the National Mathematical Modeling Competition (a tendency to idolise those who are strong)*. When I was with him, I felt he was very gentle, and we all developed a sense of admiration and closeness towards him. Getting to know him, I found he has a *rich inner world and is an interesting person*. I wanted to learn more about him and get closer to him. Being with him always allowed me to discover new things instead of just superficially looking at a face. This kind of attraction is much more lasting and profound. “(Gentle and reassuring, with an appealing presence)	Demonstration of capability signals
“People with *bad tempers* may have tendencies *toward violence (Violent* tendencies diminish a sense of security). I have a zero rating for those with tendencies toward *domestic violence*. People with *violent tendencies* are prone to causing trouble.” (Individuals with violent tendencies and unstable personalities are prone to causing trouble).	Social adaptability index
“Appearance is insignificant in the face of serious *moral flaws and personality issues*. A *good reputation* (such as being *skilled, reliable, and upright*) will greatly increase attractiveness. This represents long-term accumulated trust and is the most valuable social asset.” (Reputational capital fosters appeal and inspires trust.)	Social adaptability index
“Yes. If I hear *negative social evaluations* about a person, even if he has a good appearance, I will also start to doubt him (Negative stigma diminishes goodwill). Because these negative evaluations may reflect his *personal qualities and behavioural problems*, which will affect my trust and goodwill toward him. “(Negative stigma diminishes goodwill or trust)	Social adaptability index
Some people may have flaws in appearance, but they are *very intelligent, charming, and have high emotional intelligence (inner qualities are more important than outward appearances)*. You will unconsciously be attracted by her speech and temperament, and instead, you will think that her minor flaws become her personal characteristics, making her seem more genuine and vivid. (Enhancing one’s charm through refined speech and demeanour). Due to the limited space, only a part is shown.	Social adaptability index

The content of this table is derived from the raw interview discourse of respondents (interviews conducted according to the guidelines outlined in Appendices 1, 2). The terms enclosed in brackets following each statement represent coding labels assigned by the researcher to key expressions within the original material. Key statements have been highlighted in bold.

#### 3.4.2 Spindle code

The purpose of axial coding is to categorise and integrate subcategories formed through open coding according to their inherent logic. Researchers repeatedly examined the connotations of each subcategory, analised their interrelationships, and distilled more generalised main categories. Through continuous comparative analysis, this study synthesised eight subcategories into three main categories.

The subcategories “Youthful Health Representation,” “Pathological Risk Avoidance,” and “Genetic Expression Advantage” all point to the aesthetic psychology whereby individuals judge others’ health status through physical appearance. They are therefore grouped under the primary category “Implied Physiological Environment Health.” The three subcategories “Resource Signal Decoding,” “Spatial Safety Mapping,” and “Intergenerational Capital Transfer” all pertain to how material conditions convey security and influence attractiveness evaluations, thus falling under the primary category “Implied Safety of the Material Environment.” The subcategories “Social Status Signalling,” “Ability Worship Mechanism,” and “Social Adaptability Indicators” all reflect the shaping influence of social factors on aesthetic judgements, hence being grouped under the primary category “Implied Adaptability of the Social Environment.”

Throughout the categorisation process, researchers repeatedly cross-referenced original interview texts with subcategories, meticulously verifying the original meaning of each label to ensure classification accuracy and consistency. Through axial coding, this study established an analytical framework for understanding “face value” aesthetics across physiological, material, and social dimensions (as shown in Table 3), laying the groundwork for subsequent theoretical model construction.

**Table 3:** Spindle codes and their categories.

Main category	Subcategory	Core logic	Chain of relationships
Cue the healthiness of the physiological environment	Young Health Phenomenon	Screening mechanisms for health signals consistent with evolutionary psychology	Youthful appearance → dopamine secretion → pleasure generation
	Pathology Risk Avoidance	Defensive psychological responses triggered by disease markers	Visible pathological traits → need for safe distance → social rejection
	Gene Expression Advantage	Quality assessment systems for dominantly inherited traits	Symmetrical features/tall stature → genetic quality inferred → attractiveness bonus
Implies safety of the physical environment	Resource Signal Decoding	Physical display as a vehicle for the visualisation of competence and status	Consumption of luxury goods → demonstration of ability to acquire resources (level of personal effort) → reliability assessment
	Space security mapping	Psychological binding mechanisms of residential environment and survival security	Ownership of property → security of maintenance of offspring → weighting of long-term relationships↑
	Intergenerational capital transfer	The value of family networks implied by the stock of assets	Land/heritage → social capital visibility → ability to access resources → attractiveness premium
Implied social context adaptation	Social status signals	Capital Transformation Mechanisms for Social Status and Appraisal	Social status → resource dominance → face value premium Positive (negative) evaluations → group identification (risk of negative exclusion)
	Cult of competence mechanism	Special talents reconstruct aesthetic standards through social value mapping	Special talents → social competitive advantage → psychological activation → ‘aura of competence’ overrides physiological evaluations
	Social adaptation indicators	Individual survival advantages in a group setting through social traits such as morals and qualities	Moral Character → Cost of Trust → Benefits of Co-operation ↑ → Relationship Stability ↑

The data are mainly derived from the summary and analysis of Table 1.

#### 3.4.3 Selective coding

The third level of coding further refined the core categories. After analysis, collision, and induction, the researchers identified “the logical relationship of people’s aesthetic standards for appearance” as the core category (as shown in Table 4). The main category relationship structure was used as the “storyline,” and the phenomenon, behaviour, and context relationships were described in the form of a storyline. After completing the description of the storyline, a new theoretical framework was essentially presented.

**Table 4:** Logical relationship of categories.

Logical relation structure				Connotation of relationship structure
Physiological Environmental Characterisation	Wellness	Visual intuition generates pleasure	Increased attractiveness and value	It is believed that a beautiful and attractive appearance is healthy. A healthy body, face, genes and other physiological environment will bring people a pleasant mood and increase the sense of closeness, thus improving attractiveness. On the other hand, scruffy, diseased or unnatural damage can cause fear or repulsion.
	unhealthy	Visual intuition creates repulsion	Reduced attractiveness and lower face value	
Physical environment characteristics	rich	Indirect reflection of individual competence and endeavour	Increased attractiveness and value	Material wealth is a reflection of an individual’s material environment, and a good material environment can give people a sense of security. The material environment is to a certain extent a reflection of an individual’s ability to obtain resources, and to a certain extent it can increase a person’s attractiveness. On the contrary, a lack of material environment will indirectly make people be labelled as lazy.
	destitute	Indirect reflection of individual competence and endeavour	Reduced attractiveness and lower face value	
Social environment characteristics	harmonious	Comfort with each other increases adoration	Increased attractiveness and value	Personal qualities such as talent, character, spirituality and morality are highly contagious, and a high level of comfort and admiration can be gained by spending time with people who have a high level of overall personal qualities. People with very poor qualities and a lack of morals may bring social conflicts and potential security threats.
	unharmonious	Discomfort with each other increases aversion	Reduced attractiveness and lower face value	

The data are mainly derived from the summary and analysis of Table 1.

**Figure 3:**
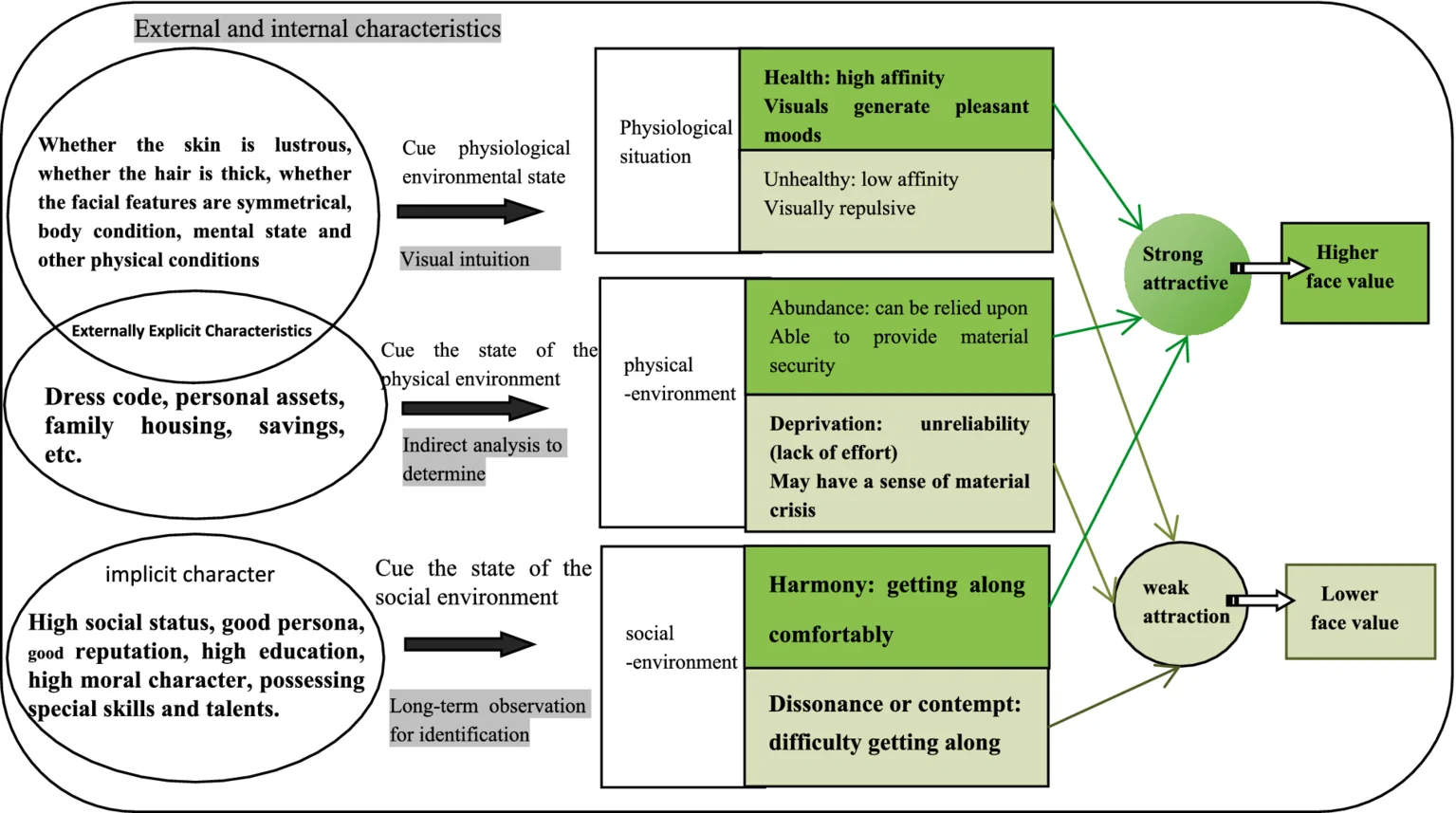
Environmental cue model within the aesthetic logic of ‘face value’.

From the data analysis, this study identified the core category, namely “the mechanism of environmental implication in the aesthetic logic of people’s ‘face value’“. From this core category, a coherent storyline emerged, centred on three types of environments—physiological, material, and social—which collectively exerted a significant influence on the strength of personal attractiveness. The strength of an individual’s attractiveness reflects their overall ‘face value’. While the ‘face value’ of *physical appearance* can be directly observed and judged through visual perception, the appeal of ‘*materialised* face value’ requires analysis and perception through material environmental factors such as clothing, jewellery, and property. By contrast, an individual’s ‘inner appeal’—encompassing talents, character and ethics—proved most elusive to direct perception, typically emerging only through prolonged interaction and understanding—as one respondent remarked: “I discovered he possessed a rich inner world and was an intriguing person. I wish to understand him more and draw nearer to him.” However, it is precisely the comfort derived from this ‘inner appeal’ that possesses the most enduring allure. Consequently, the comfort of the social environment became a crucial safeguard for the enduring vitality of an individual’s ***spiritual*** face value. Building upon this narrative thread, this study constructed a novel aesthetic logic framework for ‘face value’, summarised as the “Environmental Cue Model within the Aesthetic Logic of Face Value,” as illustrated in Figure 3.

The results of the qualitative data analysis based on multisource respondents indicate that individuals’ judgment of ‘face value’ is mainly constructed on the subjective cognitive system. Starting from the dimension of visual perception, observers tend to form cognitive judgements about the attractiveness of the target object’s appearance by systematically assessing the comprehensive environmental features it presents.

It was found that observers show a clear environmental preference when assessing appearance: they prefer healthy individuals in the physiological environment dimension; they are more likely to choose targets who display resourcefulness in the physical environment dimension; and they tend to be attracted to individuals who convey comfortable social signals in the social environment dimension. Further analyses revealed that individuals who display quality traits across multiple environmental dimensions tend to induce stronger attraction perceptions in observers. This finding provides a new empirical basis for understanding the social cognitive mechanisms underlying human aesthetic judgments.

#### 3.4.4 Theoretical saturation test

The theoretical saturation test served as the criterion for stopping sampling, specifically the point at which a category could no longer be developed because no new data were available. Therefore, in this paper, the remaining 1/4 of the study data were randomly selected for the theoretical saturation test, and coded in the same way as the previous open coding, to test for the emergence of new codes that go beyond the original category features, as exemplified below:

*I am a girl. I also enjoy looking at beautiful women. Seeing young girls with smooth, radiant skin puts me in a happy mood. My Prince Charming, in my imagination, is a ‘tall, rich, and handsome’ man—although he does not really have to be rich; being tall and handsome is already good enough. And if he knows how to be romantic and genuinely cares about people, then he’s perfect. That’s my little daydream*. (Open coding: physical appearance, genetic status, material situation, moral character).

Through careful reading and open coding of the randomly selected research data, it was found that no new concepts and categories appeared in the randomly selected research data, and the results were in line with the model of environmental cues in the aesthetic logic of ‘face value’. Therefore, the categories and theoretical models in this paper passed the theoretical saturation test.

## 4 A model of environmental cues in the aesthetic logic of ‘face value’

Following the foregoing analysis, the Environmental Cue Model within the Aesthetic Logic of Face Value effectively explains the various factors influencing an individual’s perceived attractiveness. An individual’s ‘face value’ primarily derives from three sources: (1) physiological environmental appeal, (2) material environmental appeal, and (3) social environmental appeal. This model aligns with perspectives from evolutionary psychology (e.g., Buss, 2024), gene selection theory, and sexual selection theory. Whether manifesting as overt external environmental traits or latent composite environmental characteristics, these elements collectively influence psychological perception, constituting the logical foundation for judging ‘face value’.

### 4.1 Physical surroundings can bring closeness

The aesthetic logic of aesthetic value of appearance stems primarily from the unconscious human assessment of the state of an individual’s physiological environment, which is based on a series of observable cues of physiological characteristics. Healthy physiological states are often expressed through specific phenotypic features that have significant social affinity effects in interpersonal interactions. One noteworthy finding of this study is that observers preferred people who looked young to those who were truly young. Good external maintenance (e.g., skin care, etc.) significantly enhanced observers’ aesthetic evaluations. Most respondents considered the following key dimensions in their aesthetic judgements: symmetry of facial features, skin texture, hair density, and the prominence of aging markers such as wrinkles and hyperpigmentation.

These phenotypic traits constitute important biomarkers for assessing the physiological age and health status of individuals. These explanations are consistent with evolutionary psychology (Buss, 2024), genetic selection theory and sexual selection theory, evolutionary psychology and genetic selection theory. The theory suggests that people have evolved to find certain traits attractive, particularly in terms of health, and that health is a trait that can be recognised in common by all human beings, which accounts for the ability of the human face to convey visual aesthetic signals across regions and even cultures. From an evolutionary psychology perspective, individuals with a healthy phenotype are able to induce positive emotional responses in observers, which may stem from their implied social security and intimacy potential. External features such as puffy eyes, yellowish pupils, and dull skin caused by prolonged late-night activity can reduce perceptions of health, leading observers to label individuals as having low face value. In response, people often engage in seemingly health-improving camouflaging behaviours, such as wearing makeup and receiving medical aesthetic treatments. Furthermore, physical features including cleft lip, severe malocclusion, maxillofacial deformities, or facial burn scars may elicit discomfort or fear in observers, which not only lowers perceived social attractiveness but can also trigger avoidance-oriented psychological responses. Specific biometric indicators have an important signalling value in the body size assessment dimension. Taking the WHR as an example, this indicator not only reflects an individual’s metabolic health, but is also a visual representation of reproductive potential. From the perspective of bioenergetics, an ideal body proportion implies a more efficient nutrient distribution strategy and a stronger ability to adapt to the environment. Records of height in historical documents (e.g., “Zou Ji was more than eight feet tall, and the declining sun declines in the west” in *Strategies of the Warring States*) reveal the enduring importance of this trait in the competition for mates, which is essentially a manifestation of high-quality genetic selection. In summary, the formation mechanism of physiological aesthetic preferences can be summarised into three core elements: (1) physiological soundness, (2) reproductive fitness, and (3) social security. This system of judgment reflects both the biological demands of species continuity and the complex interactions of social cognition.

### 4.2 The physical environment can bring security

In the human aesthetic evaluation system, non-physical and biological characteristics together constitute the key dimensions affecting personal attractiveness. In the multi-dimensional framework of human attractiveness evaluation, the clothing system, as an important non-verbal symbol, can significantly change the observer’s attractiveness judgment, and the difference in the evaluation of the same subject under different dressing styles confirms the substantial influence of clothing style, colour and functional design on attractiveness evaluation. Adornment and clothing not only have aesthetic modification functions, but have also evolved into symbolic carriers of personal ability, social wealth, and status symbols, and this objectification feature contributes to the individual’s strategic choice of clothing—men tend to use accessories such as high-end wristwatches and luxury leather goods to demonstrate their ability to acquire resources, and thus be labelled as “trustworthy.” Men tend to use accessories such as high-end watches and luxury leather goods to demonstrate their ability to acquire resources and be labelled as “reliable,” while women use designer bags and jewellery to map their material environment, thus forming the unique phenomenon of ‘value objectification’ in modern society. This behavioural pattern has given rise to strategic camouflage through temporary material displays (e.g., renting luxury goods) to optimise the assessment of attractiveness, essentially reflecting the functionality of physical appearance as a tool for regulating body-society relations. These visual symbol systems convey multi-dimensional messages—from social identity and value positioning to emotional attitudes—through clothing symbols such as the traditional crown or contemporary luxury goods. For example, while the traditional crown symbolises power and order, contemporary luxury goods serve as visual metaphors for economic capital. At a deeper level, the demonstrative effect of the material environment stems from the positive attributions observers make about the competence traits of resource holders. This cognitive mechanism is based on the assumption of a positive correlation between material accumulation and personal competence (e.g., business insight, decision-making judgment, and long-term effort, etc.), which allows for the dual effects of security projection and partner attraction to be generated by superior material performance. It is worth noting that this mechanism is different from shallow money worship. Its essence is to convey rational expectations of potential cooperation value and learning opportunities through material symbols rather than pure money worship, thus constructing a composite attraction assessment system unique to modern society under the interaction of biological and social choices.

### 4.3 Comfort spawned by the social environment

In a complex social network, individuals build their role competence through multiple dimensions, such as technical capital, social skills, personality traits, and moral character. The Chinese proverb “Begin with ‘face value’, get stuck in talent, and stay loyal to character” reveals the progressive evolution of attractiveness: from the initial attraction of apparent traits, to the mid-term immersion of ability and talent, and finally to the lasting attachment of moral character. This dynamic process suggests that attraction based on inner traits has significant social maintenance advantages.

Talent, as a kind of hidden capital, needs to be manifested through such concrete carriers as skillfulness, depth of knowledge or originality of thought. The interview data showed that respondents generally showed admiration and attraction to special talents (e.g., technical experts, art masters, intellectual elites), which is essentially a cognitive mapping of social competitive advantages. Through the three-level coding analysis of Grounded Theory, it was found that the social environment mainly presents a triple mechanism for constructing the source of attraction. The triple mechanism of the social environment constructing the source of attraction includes: the social harmony-oriented mechanism (i.e., the moral character and pleasantness personality enhance the relational intimacy by lowering the perception of social threat), whereas the aggression tendency triggers the social rejection due to the risk aversion instinct (e.g., “Bad-tempered people may be violent. I have zero positive feelings about people who are violent, and violent people are more likely to get into trouble with their families”); competence worship mechanism, in which individuals acquire survival capital through role modelling, and the authoritative attractiveness of higher-order competence can lead observers to reconstruct their ‘face value’. The dynamic mechanism of charisma is a kind of dynamic ‘face value’ constructed by the characteristics of passion, drive, eloquence, vision, sunshine, self-confidence, and response to others, such as emotional contagiousness, visionary leadership, etc. This kind of dynamic attraction can produce more lasting and profound interpersonal influence than static appearance, where the symbolic capital phenomenon enables a deeper understanding of traditional biological characteristics and represents a paradigm breakthrough from traditional biometric attractiveness.

## 5 Conclusions and implications

The findings of this study are significant in the following three aspects: first, health is an intuitive and key indicator of the quality of the physical environment. In today’s society, people’s lifestyles are becoming more and more complex and changeable, and many bad habits, such as alcoholism, staying up late, smoking, and long-term intake of unhealthy food can easily lead to a series of non-healthy characteristics. Therefore, people should fully recognise the importance of the physiological environment in the field of aesthetics. Make-up and cosmetic surgery can only be used as a temporary disguise for the pursuit of external beauty, while health is the core external characteristic that can truly reflect an individual’s ‘face value’. Secondly, material wealth is an important indicator of the material environment, and is also a visual reflection of personal ability or family strength, which can be regarded as a kind of materialised ‘face value’. However, excessive display of material wealth does not necessarily build trustworthy social relationships. Thirdly, the talent, character and personality of an individual are the key factors in measuring whether the social environment is comfortable. People with good character, high integrity, and gentle hearts are more likely to win the trust of others and become their followers. They are also usually able to create a safe and comfortable dating environment by virtue of their own personal charm. As *The Economist states*, for a leader to reach the top of his career, his appearance (height, muscles, tone of voice, etc.) is as important as his personal achievements. On the other hand, a person who lacks a sense of responsibility, who is not trusted by others, and who has a bad reputation will lose his charisma, just like someone who has lost his ‘soul value’, because he lost his celebrity aura due to a crime. In interpersonal interactions, although the appearance of attractiveness in the short-term interaction has a greater impact, but with the depth of interaction. When the depth of the individual’s inner soul, the beauty of the external appearance is only the icing on the cake. People will eventually grow old; only the personal ability and charisma of the ‘soul value’ can last forever.

## 6 Research limitations and future prospects

This study employed a Grounded Theory methodology, utilising in-depth interviews with 35 participants to reveal a threefold environmental suggestion mechanism underlying aesthetic judgements of physical attractiveness. Nevertheless, certain limitations remain. Firstly, we acknowledge the uneven racial distribution within the image sample. Asian faces predominate in this study, while European faces are underrepresented. Although intra-racial comparisons may mitigate interference from the outgroup effect, the theory’s universal applicability warrants further validation. Subsequent research should employ balanced samples across ethnicities to examine whether aesthetic preferences across racial groups align with the present findings, thereby testing the theory’s universality. A second limitation is the inability to precisely quantify the contribution weights of each factor, as Grounded Theory excels in theoretical construction but cannot accurately determine the relative contributions of physiological, material, and social factors to attractiveness. Subsequent research may employ quantitative methods for validation: for instance, developing scales based on subcategories, collecting data through large-scale questionnaire surveys, and then using structural equation modelling to examine the influence pathways and weighting differences of the three factors on attractiveness. Furthermore, future studies could simulate experiments in diverse social contexts (such as workplace, romantic relationships, or social networking) to investigate the moderating effect of situational factors on aesthetic weighting, thereby refining the theoretical framework.

The ‘Environmental Cue Model within the Aesthetic Logic of ‘Face Value’ constructed in this study offers a novel theoretical perspective for understanding aesthetic judgements of physical attractiveness, revealing three interrelated mechanisms: physiological, material, and social. However, as an exploratory study, its conclusions warrant further empirical validation. Subsequent research is encouraged to consolidate and refine this theoretical framework through more balanced sample designs, the incorporation of quantitative methodologies, and comparative analyses across diverse contexts.

## Data Availability

The raw data supporting the conclusions of this article will be made available by the authors, without undue reservation.
