# Supplementary material for: How environmental cues shape human face value perceptions—an exploration based on grounded theory
Source: Front Psychol. 2026 Aug 6;17:1712907. doi: 10.3389/fpsyg.2026.1712907 (PMC13491643; doi:10.3389/fpsyg.2026.1712907)
Supplement: Supplementary file 1 [file Supplementary_file_1.docx]

**Annex 1:Outline of an interview on face value aesthetics**

Dear Respondent:

Greetings! Thank you for your participation in this study on face value, aesthetics and social perception.

In today's society, ‘face value’ is often a hot topic of discussion - whether it is the evaluation of appearance on social media or the first impression of others in daily life, aesthetic standards seem to be everywhere. But does face value really depend only on features and body shape? Do factors such as social status, dress, talent and personality quality also influence our judgement of beauty?

| **Informed consent** | |
| --- | --- |
| **Research Topic:**  How Environmental Indicators Shape Human Perception of Facial Value - An Exploratory Study Based on Grounded Theory | |
| **Research institution**: Joint research team consisting of Qianan National Normal University and Catholic University of Korea, etc. | |
| **Survey Content and Objectives:**  Dear Sir/Madam:  We sincerely invite you to participate in an academic research. Before you decide whether to join this research, please carefully read the following content to fully understand the purpose, process, potential risks, benefits, and your rights of this research. You have the right to raise any questions at any time.  This research aims to explore which factors will influence people's aesthetics, such as physiological conditions, clothing and appearance, body movements, or social status, and how these factors affect people's judgment and perception of others' physical attractiveness. The duration of this research interview is approximately 10-30 minutes. If there is an emergency during the interview, you can pause or exit at any time.  During the interview or discussion, the researchers will show you some visual or textual materials (such as pictures, scene descriptions, etc.) and ask for your opinions, feelings, and evaluations.  Please directly fill in your opinions in the questionnaire. If it is a voice interview, your call will be recorded to ensure the completeness and accuracy of the information. The recording will only be used for research analysis and will be strictly kept confidential. | |
| **Potential Risks and Discomfort**  The risks involved in this study are extremely minimal. Some questions may trigger your thoughts about personal aesthetics, social comparisons, or consumption habits, which may cause mild psychological discomfort. You have the right to refuse to answer any questions at any time, or to suspend or withdraw from the study at any time, and you will not be subject to any adverse consequences. | |
| **Confidentiality**  All interview recordings and textual materials will be anonymized. Information such as your name and contact details that can identify you will be encoded instead and will not appear in any research reports, papers, or public presentations. All research data will be encrypted and stored securely on a safe server, accessible only to the research team members. After the research is completed, the original audio recordings will be retained for a full 3 years after the publication of the paper and then destroyed. | |
| **Participant's Declaration**  I have carefully read and understood the above description of the research project titled "How Environmental Clues Shape Human Facial Value Perception". All my questions have been satisfactorily answered by the researchers. I am aware that my participation is voluntary and I agree to take part in this study. | |
| Author's signature: | Interview Date: |
| (If it is a telephone interview, please simply reply "I am aware of it and agree to participate in the study") | Researcher's Signature： |

Your answers will help us to better understand the concept of beauty in modern society. The questionnaire is anonymous, and all data are for academic research only, so please fill in the questionnaire according to your own thoughts.

Face value is not only what the eyes see, but also what the mind feels.

All the pictures were randomly generated by AI and will not contain any personal information. Please feel free to give your evaluation.

We look forward to your valuable opinions! Some of the pictures in the chart may cause you discomfort, if this exists, please skip the chart!

1.Assignment question: Please rate the face value of group A B C D E on a scale of 1-5, representing very low face value, low face value, average, high face value and very high face value.

1

2

3

4

A	B	C	D	E

A （）

B （）

C （）

D （）

E （）

1.1 Can you give some reasons for the rating?

2.Who do you think has the better face in the two pictures?

 A1	B1	A2	B2

A 3	B3	A4	B4

A1（）	B1（）

A2（）	B2（）

A3（）	B3（）

A4（）	B4（）

2.1 Please briefly describe the reasons why you recognised that you chose the target to have a higher face value?

3.Who do you think has better looks in the two pictures?
2A1	2B1	2A2	2B2

2A1 （ ）	2B1（ ）

2A2 （ ）	2B2（ ）

3.1 Can you briefly describe the reasons for your choice?

4.Which weight range of the girls in the pictures below do you think has the highest face value?

42.5 Kg （ ）

51.0 Kg （ ）

55.0 Kg （ ）

61.5 Kg （ ）

68.0K g （ ）

80.0 Kg （ ）

4.1Could you briefly explain the reasons for your choice?

5.Which of the people in the pictures below do you think you like more?

1 （ ） 2 （ ） 3（ ） 4（ ） 5（ ） 6（ ） 7 （ ）

8 （ ） 9 （ ）

5.1 Can you briefly describe the reasons for your choice?

6. Who do you think has a better face in the different outfits below?

4A1	4A2

7.Do you think that apart from looks, social resources (material wealth, dress) and personal qualities (moral character, personality, character, professional skills such as music, sports, high education, title and social status) can influence a person's face value? Have you ever liked someone because of their speciality or character? For example, sing particularly well? Dance particularly well? A particularly motivated personality? Kindness? Gentle? Rich?

**Annex 2: Outline of an Interview on Facial Value Aesthetics**

Hello! Annex 2 is an in-depth interview conducted following a preliminary discussion of Annex 1, aiming to understand how people form judgments about others' appearances in daily life. There are no right or wrong answers in this interview—we are only interested in your genuine thoughts and feelings. All responses will be kept strictly confidential and used solely for academic research. If you agree, shall we begin?

Interview Questions:

1：When you first meet someone, which facial features do you notice first? (Guidance: skin, eyes, facial features, etc.)

2：What kind of facial features make you think this person is "very healthy" or "energetic"? Conversely, what features give you the impression of being "unhealthy" or "tired"?

3：Risk avoidance: If a person has noticeable skin issues (such as severe acne or scars) or facial injuries, how would you feel? Would it affect your willingness to interact with them? Why?

4：Which facial features (e.g., large eyes, a high nose bridge, double eyelids, a small mouth) do you find more attractive? Do you think these features might be associated with good genes or health?

5：Body preference: What type of body figure do you prefer? Tall, curvaceous, slim, or athletic? What feelings do different body types evoke in you? (e.g., sense of security, sexiness, health, vitality)

6.How does a person’s way of dressing (style, color, whether it’s branded) influence your evaluation of their appearance and attractiveness? Could you provide examples? (e.g., wearing red or black vs. white or pink, and the different impressions they create)

When you see someone driving a luxury car, wearing an expensive watch, or expensive jewelry, does this affect your perception of their personal charm? In what way?

If you see that a person’s home (or room) is beautifully decorated and very clean, or if you know they own property, does this change your perception of their external image? Would you find them more attractive?

When you learn that a person has a high level of education (e.g., a Ph.D.) or a respected profession (e.g., doctor, professor), do you find them more pleasant-looking or attractive? Why?

How does a person’s demonstrated talents (e.g., playing the piano, painting) or professional skills (e.g., being highly skilled at car repair) enhance their overall image and attractiveness in your eyes?

Wealth and ability: Do you admire "second-generation rich" more or "self-made wealthy people" more? Why? Does the way wealth is acquired influence your judgment of their personal qualities and attractiveness?

If you hear negative social evaluations about a person (e.g., rumors that they have a bad temper, violent tendencies, gamble, or use drugs), even if they are physically attractive, would this completely change your opinion? Conversely, would a good reputation enhance their attractiveness?

Among all the factors we’ve discussed (appearance, health, wealth, status, talent, etc.), which ones have the greatest impact on your judgment of whether someone is "attractive"?

Why do you think we are unconsciously attracted to people who appear healthy, wealthy, or talented? What needs might this reflect? (e.g., need for security, quality genes, resource assurance, or social learning)

"Our interview is now complete. The perspectives you shared are incredibly valuable and highly significant for our research. Once again, thank you sincerely for your time and honest contributions! We wish you all the best in your daily life!"
